# Dive-by-dive variation in the diving respiratory air volume of southern elephant seals (*Mirounga leonina*)

**DOI:** 10.1242/jeb.249659

**Published:** 2025-05-23

**Authors:** George Sato, Taiki Adachi, Christophe Guinet, Patrick Miller

**Affiliations:** ^1^ Sea Mammal Research Unit, University of St Andrews, St Andrews KY16 8LB, Scotland, UK; ^2^ National Institute of Polar Research, Tachikawa, Tokyo 190-8518, Japan; ^3^ CNRS Centre of Biology Studies of Chizé, 79360 Villiers-en-Bois, France

**Keywords:** Phocid seals, Hydrodynamic glide model, Buoyancy, Stroke intensity

## Abstract

The role of diving respiratory air volume (DRAV) in deep-diving phocid seals remains poorly understood, largely because of the lack of methods for measuring DRAV in free-ranging divers that exhale before diving. We developed a method to estimate DRAV using a hydrodynamic glide model applied to descent glides recorded using multi-sensor data loggers. We estimated dive-by-dive DRAV for six negatively buoyant female southern elephant seals (*Mirounga leonina*). During shallow descent glides, rapid compression of DRAV influenced net buoyancy and gliding speed, making this phase suitable for estimating DRAV. Our results revealed dive-by-dive variation in DRAV, which was positively correlated with root mean square (RMS) sway acceleration (a proxy for per-stroke effort) and the depth at which gliding began during the initial descent. DRAV increased with both tissue density and maximum dive depth, suggesting that seals adjusted their DRAV to stay closer to neutral buoyancy through their dives. However, the observed level of adjustment did not result in neutral buoyancy at half of the maximum dive depth, as predicted to minimise round-trip locomotion costs. Instead, the seals typically adjusted DRAV to reach neutral buoyancy at ∼30 m depth, <10% of their mean maximum dive depth. This indicates that strong negative tissue density imposes transit costs that cannot be fully compensated for by DRAV adjustment alone. Future work should explore whether other breath-hold divers show similar patterns of DRAV adjustment and quantify the associated physiological and ecological benefits.

## Teaser

**Summary:** Elephant seals adjust their diving respiratory air volume (DRAV) depending on their tissue density and maximum dive depth. Adjusting DRAV regulates their net buoyancy during dives.

## INTRODUCTION

Natural selection has led to a range of physiological and behavioural adaptations in breath-hold divers to minimise energy use during a dive, because of the fitness benefits of maximising their time foraging underwater (Thompson and Fedak, 2001). The time animals can remain underwater during a dive is considered to be largely determined by their aerobic dive duration – the maximum duration a diving animal can remain submerged while relying on aerobic metabolism (Kooyman et al., 1983). This aerobic duration is limited by both the amount of available oxygen and the rate at which it is consumed.

In order to extend their time underwater, deep-diving animals can reduce their oxygen consumption. This reduction is associated with a ‘dive response’, which is achieved through selective blood redistribution from digestive organs and muscles to critical organs such as the brain and heart, coupled with a decrease in heart rate and a reduction in body temperature during extreme dives (Andrews et al., 1997; Kooyman and Ponganis, 1998; Williams and Ponganis, 2021). These animals can also conserve oxygen by optimising energy expenditure through adjusting their swimming gaits, switching between prolonged glides, stroke-and-glides or continuous stroking (Richard et al., 2014; Williams, 2001; Williams et al., 2004, 2000). These physiological and behavioural adaptations, which include reallocating oxygen to vital organs and switching swimming gaits, are key in species like phocid seals, especially elephant seals (*Mirounga* spp.), which spend a large proportion of their time at sea conducting deep dives.

Swimming costs are influenced by the diver's net buoyancy (Adachi et al., 2014; Aoki et al., 2011; Cole et al., 2023; Miller et al., 2012; Piot et al., 2023; Richard et al., 2014; Sato et al., 2002; Trassinelli, 2016; Watanabe et al., 2006). During foraging dives, field studies have shown that seals glide in the buoyancy-aided direction, reducing the one-way cost of transport (metabolic cost per unit mass per unit distance; Schmidt-Nielsen, 1972), while stroking in the buoyancy-hindered direction to overcome external forces (Williams et al., 2000). Buoyancy has a significant impact on swimming costs and, consequently, overall energy expenditure during foraging for elephant seals (Maresh et al., 2014; McIntyre et al., 2010). Studies have shown that round-trip swimming costs for elephant seals increase when they deviate from neutral buoyancy (Adachi et al., 2014). For instance, a 1% change in tissue density can lead to a 15% increase in ascent swimming effort (Richard et al., 2014). This is because the seal maintains the same swimming speed in the buoyancy-hindered direction regardless of tissue density, possibly a strategy to transit at their optimum speed to minimise swimming costs (Watanabe et al., 2011). As the metabolic rate during gliding is already close to its basal level, any increase in energy expenditure while swimming in the buoyancy-hindered direction cannot be fully compensated for by gliding in the aided direction, leading to higher overall costs for non-neutrally buoyant seals (Miller et al., 2012).

Given the substantial influence of net buoyancy on round-trip swimming costs, it is reasonable to predict that breath-hold divers have evolved behavioural strategies to adjust their buoyancy to minimise energy expenditure. While tissue density has been extensively studied for its role in influencing round-trip swimming costs, it reflects long-term foraging success and cannot be altered on a dive-by-dive basis. Diving respiratory air volume (DRAV), in contrast, has the flexibility to be adjusted. However, there has been limited research on how deep-diving animals, such as elephant seals, use DRAV to manage buoyancy and energy expenditure during their dives.

For shallow-diving animals that rely on DRAV as a major component of their total oxygen store, such as penguins, studies have shown that they adjust DRAV according to dive depth to maximise oxygen storage and extend the aerobic capacity during deep dives (Ponganis et al., 2011; Sato et al., 2002). While increasing DRAV elevates the total oxygen store, penguins appear to reduce their DRAV during shallower dives to expend less energy, allowing them to overcome buoyant forces at shallow depths (Sato et al., 2011, 2002). Additionally, an experimental study on Steller sea lions (*Eumetopias jubatus*) has also suggested that breath-hold divers may adjust DRAV to maintain their diving metabolic rate, compensating for changes in net buoyancy (Fahlman et al., 2008).

In contrast, deep-diving animals are thought to rely much less on DRAV as part of their total oxygen store to reduce the risk of decompression sickness (Fahlman et al., 2009). However, DRAV still plays a significant role in their locomotion, especially at shallow depths where DRAV is less compressed. For instance, even though DRAV makes up only a small portion of the total oxygen store in sperm whales (*Physeter macrocephalus*), they exert forceful downward strokes during the initial phase of dives to counter buoyancy, then glide during the final phase of ascents (Miller et al., 2004). Similarly, Blainville's beaked whales (*Mesoplodon densirostris*) reduce their stroking effort while increasing speed in the first 30 m of descent as DRAV is compressed, indicating reduced effort at greater depths (Martín López et al., 2016). The optimum swim speed of short-finned pilot whales (*Globicephala macrorhynchus*) is also influenced by buoyancy due to the expansion of DRAV (Aoki et al., 2017). These observations suggest that DRAV plays an important role at shallow depths, potentially affecting round-trip swimming costs particularly during the early descent and late ascent, where its influence on buoyancy is strongest. However, the factors that influence DRAV variation among deep divers have not been fully explored.

Trassinelli's (2016) theoretical study explored strategies for minimising swimming costs by examining the interaction between tissue density and DRAV. In Trassinelli's (2016) model, tissue density remains constant with depth, while DRAV compresses following Boyle's Law as depth increases as a result of rising hydrostatic pressure. For purely vertical dives, the benefit of adjusting DRAV depends on the tissue density of the animal. The optimal cost-minimising strategy for a neutrally buoyant seal is to reduce DRAV to zero, as this allows the animal to maintain minimal thrust effort throughout the dive (Trassinelli, 2016). Seals closer to neutral buoyancy have been observed to use a stroke-and-glide swimming pattern throughout their dives, in contrast to the prolonged glides and continuous stroking seen in negatively buoyant seals, where buoyancy aids or hinders movement (Sato et al., 2013). For animals with negatively buoyant tissue density, swimming costs are predicted to be minimised when they maintain a DRAV that achieves neutral buoyancy at approximately half of the maximum dive depth (Trassinelli, 2016). The effect of residual DRAV on net buoyancy has often been overlooked for exhaling divers, as it was considered negligible (Gallon et al., 2007). However, exhaling before diving can reduce thrust effort, especially when tissue density is close to neutral buoyancy. While exhaling before a dive is often seen as a mechanism to prevent decompression sickness by inducing alveolar collapse (Hooker et al., 2005), it may also affect swimming costs. This raises the question of whether phocid seals, which exhale before breath-hold dives, regulate DRAV not only to manage decompression risk but also to minimise swimming costs.

The aim of this study was to estimate DRAV from descent glides and investigate whether elephant seals adjust their DRAV in response to dive depth and tissue density to reduce swimming costs. Based on Trassinelli's (2016) theoretical framework, we predicted that: (1) seals reduce DRAV as their tissue density approaches neutral buoyancy, and (2) negatively buoyant seals increase their DRAV on deeper dives, enabling them to achieve neutral buoyancy at greater depths, minimising overall swimming costs.

## MATERIALS AND METHODS

### Field site and study animals

Animal-attached movement data were collected from six, post-breeding female southern elephant seals, *Mirounga leonine* (Linnaeus 1758) (Goulet et al., 2020, 2019; Jouma'a et al., 2017). The seals were tagged at Kerguelen Islands. Tagging occurred in October 2017 (seals ml17_301 and ml17_280), in October 2018 (ml18_292, ml18_294a, ml18_294b) and in October 2019 (ml19_295a). Each seal was anaesthetised and then equipped with a head-mounted DTAG-4 sound and movement tag (97×55×33 mm, 200 g in air) as well as a neck-mounted Argos tag (SPOT-293, Wildlife Computers, 72×54×24 mm, 119 g in air). The tags were retrieved when the seals returned to moult in December and January. Full details on animal handling and the tagging process can be found in Goulet et al. (2020, 2019). Ethical approval was provided by the French Committee for Polar Environment and the University of St Andrews Animal Welfare and Ethics Committee.

### Dive analysis

Tag-data analysis was conducted in Igor Pro 8 (WaveMetrics, Inc., Lake Oswego, OR, USA) and MATLAB (MathWorks, Inc., Natick, MA, USA). The tag sampled high-resolution depth (25 Hz) and triaxial acceleration (200 Hz) for over 30 days (Goulet et al., 2020, 2019). To capture long-term, independent changes in buoyancy (i.e. tissue density), each 24 h record was spaced at least 6 days apart to ensure independent tissue density estimates.

For the analysis, the depth and triaxial acceleration data were down sampled to 1 Hz and 25 Hz, respectively. Speed (*U*) was calculated as the change in depth per unit time (seconds) divided by sin(pitch) for pitch angles greater than |30 deg| (Miller et al., 2004).

A dive was defined as when the animal dived deeper than 5 m depth (>5 m), and a glide was defined as the passive movement of an animal through a medium. Each dive was segregated into four distinct phases: surface, descent, bottom and ascent phases. The surface phase was when the seal was shallower than 5 m. As defined by Miller et al. (2004), the start of the descent phase was recorded when the animal left the surface and dived deeper than 5 m depth. The bottom phase started when the pitch angle of the seal first exceeded 0, and the ascent phase started at the last point in time when the pitch angle was negative. As all the elephant seals were negatively buoyant, this study focused on using the descent phase, particularly shallow descent glides, to estimate DRAV at the start of each dive.

### Stroke analysis

Flipper movements, or strokes, were defined as side-to-side oscillations, and one stroke cycle refers to the completion of one complete flipper oscillation. The stroking pattern was measured using triaxial dynamic acceleration in the body frame, which includes surge (anterior–posterior axis), sway (left-to-right axis) and heave (dorsal–ventral axis) accelerations. Given that phocid seals generate propulsion through lateral (side-to-side) body undulations, sway acceleration was used to detect strokes. The accelerometer recorded both gravity-based acceleration (reflecting body orientation) and dynamic acceleration (reflecting stroking) (Sato et al., 2003). A low-pass filter was applied to the measured acceleration of each axis to separate the gravity-based acceleration from the dynamic acceleration. The filter frequency was determined from the power spectral density of the sway acceleration. Following Adachi et al. (2023), gravity-based acceleration was used to calculate the pitch angle. Dynamic acceleration was filtered out by subtracting gravity-based acceleration from the total measured acceleration (Sato et al., 2004, 2003). The resulting high-pass filtered acceleration was used to detect strokes and glides, with stroke detection thresholds specific to each individual. Stroke intensity was quantified using the root mean square (RMS) of dynamic acceleration over a half-stroke (the period between two consecutive zero-crossings). To compare per-stroke effort across varying levels of buoyancy, the mean RMS across all half-strokes was calculated for the descent phase between 5 and 30 m depth that included at least three complete half-strokes. These mean RMS values were then used to assess correlation with estimates of DRAV.

### Hydrodynamic glide model

**Fig. 1.**
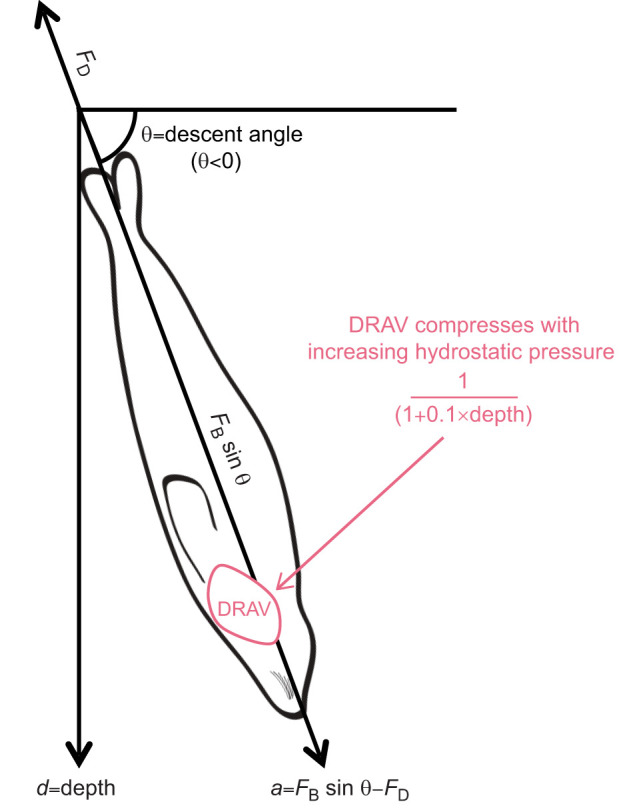
**Scheme of the forces acting on a descending elephant seal.** Here, the seal is descending with an angle θ. Drag, *F*_D_, acts opposite relative to the movement of the animal. Buoyancy, *F*_B_, acts vertically so the effect of buoyancy on the acceleration of the animal is weighted by sinθ. The net buoyancy accounts for the air (diving respiratory air volume, DRAV) and non-air components (tissue density). DRAV compresses with increasing depth as a result of increasing hydrostatic pressure, while tissue density was assumed to remain constant over shallow depths.

The total external forces acting upon a passively gliding animal can be estimated from the difference between drag (*F*_D_) and buoyancy (*F*_B_) as *F*_B_sin(*p*)−*F*_D_=*ma*, where *m* is the animal's mass, *a* is the acceleration and *p* is the pitch of the animal. Buoyancy changes with the sine of pitch (see Fig. 1). The full expression of the forces affecting the acceleration of a gliding animal can be estimated using the hydrodynamic glide model from Miller et al. (2016) (Eqn 1):
(1)

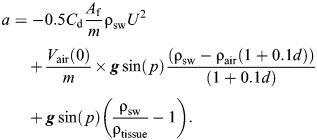
Eqn 1 comprises three terms representing the external forces that influence acceleration: drag, DRAV and tissue density relative to the surrounding seawater. The first term represents drag, which acts opposite relative to the movement of the animal. This term contains measured parameters such as the seawater density (ρ_sw_, kg m^−3^), the measured speed of the animal (*U*, m s^−1^), frontal surface area (*A*_f_, m^2^) and mass (*m*, kg).

Given that seawater density varies with depth due to changes in temperature and pressure, ρ_sw_ was modelled as a function of depth [ρ_sw_(*d*)]. To account for this variation, ρ_sw_ was calculated for each depth measurement during glides. Seawater temperature and salinity data were obtained through the World Ocean Database (https://www.nodc.noaa.gov/OC5/SELECT/dbsearch/dbsearch.html) (Boyer et al., 2018). Mass and girth of each animal were measured prior to tag deployment. Frontal surface area (*A*_f_) was calculated from girth (*G*, m) using the equation *A*_f_=π(*G*/2π)^2^. The remaining unknown term is the drag coefficient (*C*_d_), which was estimated from simulating the terminal gliding speeds across a range of *C*_d_ values (0.01–1) (Adachi et al., 2023). Terminal gliding speed occurs when drag equals buoyancy during extended descent glides (Aoki et al., 2011). The best-fit *C*_d_ was estimated by minimising the mean square error (MSE) between observed and simulated terminal speed. The median best-fit value across individuals was 0.03, with a range of 0.02–0.05, and this value was used in this study. *C*_d_ was assumed to remain constant across dives, as its dependence on Reynolds number (*Re*) stabilises at high *Re* (Vogel, 1994).

The second and third terms represent the net buoyancy force, defined as the difference between the mass of the displaced fluid and the mass of the animal. The body can be divided into air-filled spaces (DRAV) and non-air (tissue density) components (Miller et al., 2016). The second term accounts for the buoyant forces generated by DRAV, which compresses with increasing depth from increasing hydrostatic pressure according to Boyle's law. This term includes the gravitational constant, ***g*** (9.81 m s^−2^), depth (*d*, m), seawater density (ρ_sw_, kg m^−3^), air density at the surface (ρ_air_, 1.225 kg m^−3^ at 101,325 Pa and 15°C) and the pitch angle of the animal (*p*, deg). *V*_air_(0) is the volume of air carried from the sea surface (ml).

The third term reflects the effect of tissue density (ρ_tissue_, kg m^−3^) on net buoyancy. Tissue density (ρ_tissue_) was estimated by solving the hydrodynamic glide model using the obtained drag coefficient (*C*_d_=0.03). To ensure independent estimation of DRAV and ρ_tissue_, ρ_tissue_ was calculated using data from glides deeper than 100 m, where the effect of DRAV is negligible because of the high hydrostatic pressure, thereby excluding the second term from the hydrodynamic glide model to solve for ρ_tissue_ (Aoki et al., 2011; Biuw et al., 2003). This study then used values of *C*_d_ and ρ_tissue_ derived from deep gliding periods (Adachi et al., 2023) to estimated DRAV during shallow glides, where its contribution to net buoyancy remains substantial.

Previous applications of the hydrodynamic glide model have assumed that animal tissues are incompressible in seawater (Miller et al., 2004). Although studies suggest that incorporating tissue compressibility can improve model accuracy, its overall effect remains minimal. For instance, the body of northern bottlenose whales were found to compress by only 0.3% at depths of 1000 m (Miller et al., 2016). Given that the present study focused exclusively on gliding periods at shallow depths, tissue density ρ_tissue_ was assumed to remain constant throughout each glide.

Additionally, the hydrodynamic glide model does not account for induced drag generated by lift-producing appendages such as flippers (Sato et al., 2002). Lift is generally considered to be useful when an animal maintains a non-vertical or horizontal posture to counteract gravity during a dive (Sato et al., 2013, 2002). However, this study specifically targeted steep descent phases, during which the effect of lift and thrust-induced drag is expected to be negligible (Aoki et al., 2011).

### DRAV estimation

During the initial phase of descent, animals forcefully stroke downwards to counteract drag and net buoyancy (Martín López et al., 2015, 2016; Miller et al., 2004). They continue to swim steadily until the point at which positive buoyancy forces from DRAV are overwhelmed by the increasing hydrostatic pressure exerted on the animal, at which point the gliding phase begins. Assuming that the compressibility of the trachea and of the lungs reflects a perfectly compliant air space following Boyle's law *V*_air_(*d*)=*V*_air_(0) /(1+0.1×*d*), then gliding acceleration is influenced by changes in DRAV as the seal dives deeper. Therefore, DRAV was estimated by modelling the changes in speed during the glide.

**Fig. 2.**
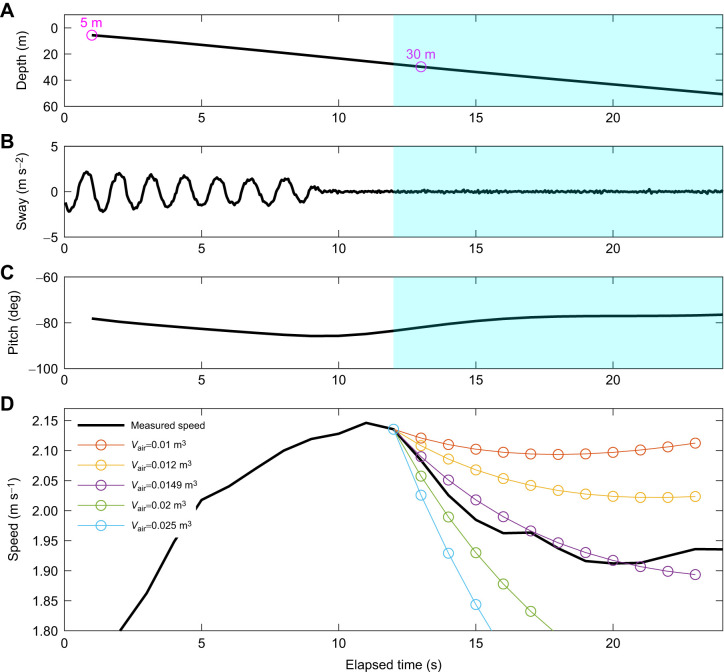
**Speed simulation and DRAV estimation from descent glides.** (A) Depth profile of the shallow descent phase. Note that dives where the gliding phase (blue shading) initiated before 30 m depth were selected for analysis. (B) Flipper stroking behaviour indicated by the oscillations in the sway acceleration. (C) Descent pitch angle of the animal. (D) The relationship between measured speed (thick black line) and simulated speed with different DRAV values (coloured circles and lines). In A–C, the non-shaded area represents the stroking period, while the blue shaded area represents the gliding period of the animal. The start of the simulated speeds corresponds with the start of the gliding period. The air volume (*V*_air_) with the best model fit in this example was 0.0149 m^3^.

Glide segments shallower than 50 m were selected, and swim speed was simulated from the start to the end of the glide by using the hydrodynamic glide model. Eqn 1 of the glide model was rewritten as:
(2)

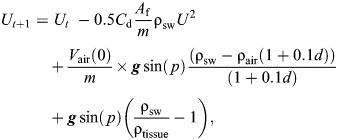
where *U_t_*_+1_ and *U_t_* are swim speeds (m s^−1^) at times *t*+1 and *t*, respectively. At *t*=0, the speed at *U_t_*_+1_ (*U*_1_) was computed by substituting measured variables [initial speed (*U*_0_), seawater density (ρ_sw_), pitch (*p*) and depth (*d*)], physical constants [gravitational acceleration (***g***), air density (ρ_air_)], and estimated parameters [drag coefficient (*C*_d_) and tissue density (ρ_tissue_)] into the model (Eqn 2). This process was iterated at 1 s intervals (i.e. *U_t_*_+1_) until the glide reached a depth of 50 m (see Fig. 2).

The speed of the gliding animal was simulated using a range of air volumes ranging from 0 m^3^ to 0.03 m^3^ with increments of 0.0001 m^3^. The upper bound (0.03 m^3^) was chosen to encompass the maximum plausible lung capacity for the heaviest animal in the dataset (417 kg), based on the measured maximum lung capacity of 50.4 ml kg^−1^ for elephant seals (Kooyman et al., 1972), while also providing flexibility across the tested range. For each candidate air volume, the following steps were performed: (1) select a candidate air volume within the range of 0 m^3^ to 0.03 m^3^; (2) simulate speed changes over the glide using Eqn 2; (3) repeat steps 1–2 for the full duration of the glide; and (4) repeat the entire process for the full range of candidate air volumes (Fig. 2).

Once the speed had been simulated for the whole range of possible air volumes, each simulated speed profile was compared with the measured speed by calculating the MSE. The MSE was weighted by a compression factor, *c*, following Boyle's law, giving more weight to glide periods near the surface where DRAV's effect on net buoyancy is more pronounced. The MSE was described as:
(3)

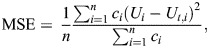
where *U_i_* is the speed for a given data point *i*, n is the number of data points within a single glide, *U* is the measured speed, *U_t_* is the simulated speed at time *t* from Eqn 2 and *c* is the compression factor at a certain depth *d*, for a given data point *i*. *c_i_* was defined as:
(4)

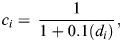
where *d_i_* is the depth for a given data point *i*. The air volume with the lowest MSE was considered to be the best estimate for DRAV for glides that satisfied the four criteria mentioned below.

DRAV was estimated from descent glides between 5 and 50 m depth that met the following criteria: (1) dive descents wherein gliding started shallower than 30 m depth; (2) dive descents with a glide duration longer than 10 s; (3) dive descents with an average pitch angle steeper than −60 deg; and (4) stable glides with a circular variance of roll and pitch less than 0.1.

Criteria 1 and 2 were important to record the deceleration from DRAV. Changes in air volume at deeper than 50 m (16.7% of surface DRAV) were not substantial enough to affect the speed of the passive glide. In order to compare the measured and simulated speed over sufficient time for forces to affect speed, only glides with a duration longer than 10 s were used. Criterion 3 was set to minimise the error in the DRAV estimation. For instance, glides with a pitch angle shallower than −60 deg may be affected by the induced drag by lift-generating organs, underestimating the DRAV for the dive. Criterion 4 was essential to extract stable glides where the animal is passively gliding during the descent phase. The dives based on these criteria yielded dives with a maximum dive depth deeper than 100 m, because the behaviour of the seals appeared to be more variable for shallower dives.

### Depth at neutral buoyancy

To evaluate how DRAV influenced buoyancy with increasing depth, we calculated the depth at which net buoyancy transitioned from opposing to aiding the animal's movement during descent. At each point during the descent, the net buoyancy force was calculated as:
(5)

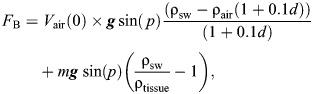
where *F*_B_ is the buoyancy force, and *V*_air_(0), the air volume at the surface, was obtained by multiplying the estimated DRAV by the seal's mass. The remaining parameters are as defined in Eqns 1 and 2. We examined each descent glide point-by-point over a depth range from 1 m to the maximum dive depth, identifying the depth at which the net buoyancy force changed sign (the point at which the buoyancy force changed from hindering to aiding) as the depth of neutral buoyancy. If net buoyancy aided the descent from the onset, the neutral buoyancy depth was recorded as <1 m. This analysis was limited to dives that included descent glides meeting the four criteria outlined in the previous section.

### Buoyancy-driven variation in swimming effort as a function of DRAV

We assessed whether variability in estimated DRAV across dives was reflected in swimming kinematics as a means of evaluating the consistency of the estimates with variation in locomotor effort.

Although elephant seals exhale prior to diving, they retain a measurable amount of residual air volume (Kooyman et al., 1972). This residual air is expected to influence descent swimming behaviour at shallow depths, as shown in other deep-diving species such as sperm whales and beaked whales (Martín López et al., 2016; Miller et al., 2004).

To evaluate the influence of residual DRAV on swimming effort during descent, we examined its relationship with two kinematic indicators: (1) RMS sway dynamic acceleration, and (2) depth of stroke cessation (i.e. the depth at which the animal transitions from stroking to gliding).

Seals with higher residual DRAV are expected to experience greater positive buoyancy near the surface, requiring increased thrust to initiate descent. This greater effort should manifest as increased RMS sway dynamic acceleration during stroking. We modelled RMS sway dynamic acceleration as a response variable, with DRAV as the explanatory variable. This relationship was examined to test whether variation in per half-stroke swimming effort corresponded with variation in DRAV. Analysis focused on half-stroke segments during descent at shallow depths (5–30 m), where the buoyant effect of DRAV is strongest. Data shallower than 5 m were excluded to avoid the effects of flipper–surface interaction. Importantly, RMS sway dynamic acceleration is independent of the variables used to estimate DRAV. DRAV was estimated from depth, vertical speed, pitch angle and sea water density. Pitch was derived from the low-pass-filtered tri-axial acceleration.

Because animals are expected to stroke until DRAV is sufficiently compressed (i.e. buoyancy reduced), we also modelled the depth of stroke cessation as a function of DRAV. This analysis tested whether seals with greater DRAV tended to stroke deeper before transitioning to gliding. In summary, we assessed the following two relationships for each dive: (A) RMS sway dynamic acceleration against DRAV, and (B) depth of stroke cessation against DRAV.

### Sensitivity analysis

The drag coefficient *C*_d_ was set at 0.03 for our analyses, but potentially varies across individuals, ranging from 0.02 to 0.05 (Adachi et al., 2023). As *C*_d_ is a key parameter in the hydrodynamic glide model, this variation could influence DRAV estimates derived from Eqns 1 and 2. To evaluate how uncertainty in *C*_d_ affected our results, we repeated the statistical models using DRAV estimates derived from four different values of *C*_d_ across the plausible range. This allowed us to assess whether the observed relationships were robust to uncertainty in *C*_d_.

### Statistical analyses

Linear mixed models were constructed in R version 4.3.2 (http://www.R-project.org/) using the nlme package (https://CRAN.R-project.org/package=nlme). To account for temporal autocorrelation, we applied the corAR(1) function to all statistical models. Each model underwent 1000 bootstrap iterations to estimate predicted parameters and 95% confidence intervals. A statistical critical value of 0.01 was applied to reduce the risk of Type I errors from multiple testing.

Two univariate linear mixed models were constructed to assess kinematic responses to variation in DRAV. In Model A, the response variable was RMS sway dynamic acceleration; in Model B, it was depth of stroke cessation. In both models, DRAV was the explanatory variable. Both models included individual as a random effect. We report both the marginal *R*^2^ (*R*^2^_m_; variance explained by fixed effects) and conditional *R*^2^ (*R*^2^_c_; variance explained by both fixed and random effects) (Nakagawa and Schielzeth, 2013).

To evaluate the effects of maximum dive depth and tissue density on DRAV, we constructed a multivariate linear mixed model. This model, distinct from those used for kinematic validation, included DRAV as the response variable with a Gaussian error distribution. Maximum dive depth and tissue density were set as explanatory variables, and an interaction term between tissue density and dive depth was included to account for their combined effects. Individual was treated as a random effect.

For model comparison with nested fixed effects (using the same random structure), linear mixed models were fitted using maximum likelihood rather than restricted maximum likelihood (Zuur et al., 2009). The nested models were compared using the Akaike information criterion (AIC) and the dredge function from the MuMIn package (https://CRAN.R-project.org/package=MuMIn). This approach facilitated selection of the best-fit model and enabled computation of summed Akaike weights to assess the relative importance of each variable across the models.

## RESULTS

Of the 1638 dives recorded from the six tagged seals, DRAV was estimated for 270 dives that met the predefined selection criteria. DRAV estimates ranged from 0 to 53.7 ml kg^−1^. A value of 0 ml kg^−1^ was derived from a dive where gliding began at a depth shallower than 10 m. As seals are unable to fully exhale their DRAV, this value probably reflects an estimation error. Excluding this estimate had a negligible effect on model estimates and variance explained, so DRAV values from 269 dives were used in subsequent analyses.

For these dives (*N*=269), seals descended at a mean (±s.d.) pitch angle of −74.9±6.6 deg with a mean speed of 1.84±0.23 m s^−1^, reaching an average maximum dive depth of 434.5±144.4 m (Table 1). Mean estimated DRAV was 21.1±8.1 ml kg^−1^ (range: 1.3 to 53.7 ml kg^−1^).

**Table 1.** Diving behaviour and diving respiratory air volume (DRAV) of the tagged Southern elephant seals (*Mirounga leonina*) in this study

Animal ID	Mass (kg)	Frontal surface area (m^2^)	Day	Number of analysed dives		Mean tissue density (kg m^−3^)	Descent pitch (deg) (*N*=269)	Descent speed (m s^−1^) (*N*=269)	Depth at stroke cessation (m) (*N*=269)	RMS sway acceleration (m s^−2^) (*N*=114)	Max. dive depth (m) (*N*=269)	DRAV (ml kg^−1^) (*N*=269)
				DRAV (*N*=269)	Stroke intensity (*N*=114)							
ml17_280a	395	0.27	2	3	3	1034.8	−62.5±2.7	1.7±0.04	28.1±0.3	0.99±0.06	457±154	20.2±5.4
			9	18	3	1036.3	−68.5±4.7	1.7±0.08	21.6±4.8	0.93±0.03	612±91	21.5±4.8
			17	22	13	1035.3	−76.6±3.2	1.7±0.08	23.4±4.6	1.05±0.14	572±65	23.6±5.5
			24	18	5	1034.2	−77.8±2.9	1.7±0.10	17.1±6.2	0.99±0.10	481±26	15.3±7.4
ml17_301a	417	0.27	2	2	2	1041.2	−63.2±2.6	2.1±0.02	26.3±1.9	1.36±0.07	505±103	51.1±3.7
			9	0	0	1039.2	–	–	–	–	–	–
			17	15	8	1037.3	−76.8±2.4	2.0±0.05	22.6±6.2	1.27±0.11	494±94	28.8±8.2
			24	25	9	1037.0	−76.9±2.4	1.9±0.11	19.4±7.1	1.16±0.17	481±86	21.8±8.7
ml18_292a	238	0.20	2	1	1	1037.1	−75.2	1.7	22.8	1.41	431	26.1
			9	0	0	1037.2	–	–	–	–	–	–
			17	3	3	1037.2	−76.8±1.1	1.6±0.09	22.8±7.1	1.31±0.18	488±73	35.3±6.1
			24	1	1	1038.9	−76.9	1.7	28.1	1.23	609	42.4
ml18_294a	238	0.20	3	0	0	1035.8	–	–	–	–	–	–
			10	14	7	1035.7	−70.7±2.7	1.4±0.15	20.8±8.1	1.16±0.10	450±109	24.0±6.1
			18	9	6	1035.6	−73.1±6.4	1.4±0.12	24.3±5.5	1.17±0.07	488±89	26.4±4.7
			25	1	0	1035.7	−76.1	1.4	29.8	–	583	27.3
ml18_294b	303	0.22	2	1	1	1036.4	−78.7	1.9	29.7	1.46	426	36.3
			9	24	9	1036.7	−78.9±4.4	1.9±0.15	15.2±6.5	1.25±0.10	327±107	20.1±6.8
			17	21	2	1036.5	−81.9±3.4	1.8±0.12	12.3±5.9	1.21±0.07	338±134	17.4±7.7
			24	18	4	1035.5	−79.6±6.0	1.8±0.09	16.0±4.8	1.15±0.18	348±186	16.6±6.4
ml19_295a	288	0.21	2	6	2	1038.2	−65.9±6.0	1.8±0.13	22.4±4.7	1.00±0.05	456±77	15.0±6.3
			9	13	7	1039.7	−65.6±4.6	1.9±0.12	17.7±5.2	1.26±0.13	430±167	24.6±7.5
			17	54	28	1039.4	−70.4±3.3	2.1±0.10	16.8±5.3	1.14±0.09	354±136	18.4±5.5
Total	–	–	–	269	114	–	–	–	–	–	–	–
Mean	313.2±76.8	0.23±0.03	–	–	–	1037.0±1.7	−74.9±6.6	1.84±0.22	18.8±6.7	1.18±0.16	435±144	21.1±8.1

Data are means±s.d. RMS, root mean square.

**Fig. 3.**
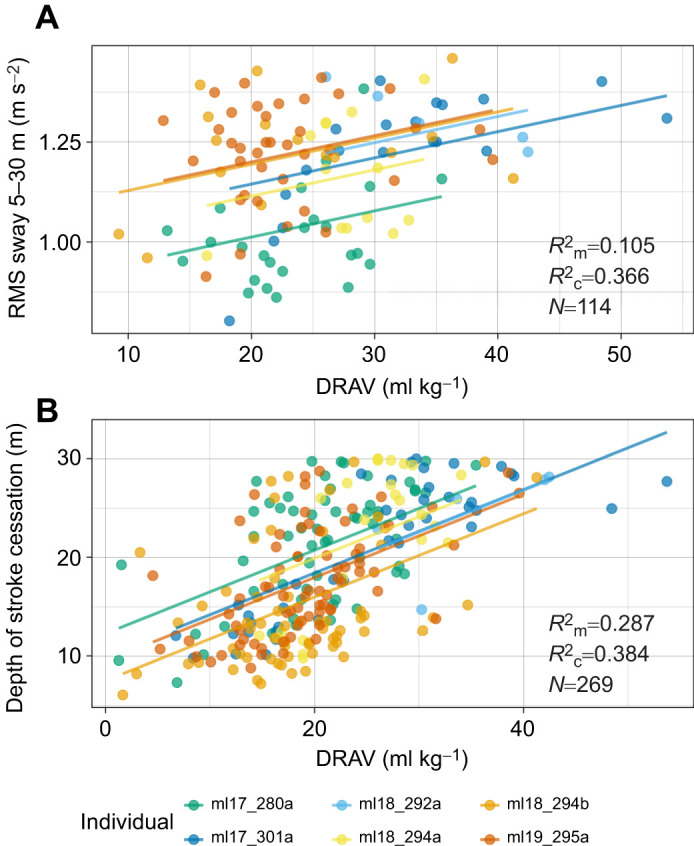
**The relationship of DRAV with RMS sway dynamic acceleration and depth of stroke cessation.** (A) Root mean square (RMS) sway acceleration, a proxy for per-stroke effort, and DRAV (*N*=114 dives, *R*^2^_m_=0.105, *R*^2^_c_=0.366, ϕ=0.243, *P*<0.001). (B) Depth of stroke cessation and DRAV (*N*=269, *R*^2^_m_=0.287, *R*^2^_c_=0.384, ϕ=0.335, *P*<0.001). *R*^2^_m_ represents the marginal *R*^2^ from the linear mixed model, accounting only for the variation from the fixed effect (DRAV). *R*^2^_c_ represents the conditional *R*^2^ from the model, accounting for both the fixed and random effect (individual variation). The corAR(1) coefficient (ϕ) is reported for both models. Note that the number of analysed dives differs across A and B as a result of the difference in the number of dives that met the required criteria. The colours represent the different individuals (*N*=6). A contains data points from dives that were able to estimate DRAV with at least three half-strokes between 5 and 30 m and hence it has the lowest number of analysed dives (*N*=114). Each point shown in A is the RMS sway dynamic acceleration per analysed dive, and thus one data point represents one dive. B contains dives where DRAV could be estimated (*N*=269), and one data point represents one dive.

Among the 269 dives where DRAV was estimated, 114 dives contained three complete half-strokes between 5 and 30 m depth. During these descents, the seals had a RMS sway dynamic acceleration of 1.18±0.16 m s^−2^ (mean±s.d.) and ceased stroking at a mean depth of 18.8±6.7 m (Table 1). DRAV showed a significant positive relationship with both RMS dynamic acceleration (*N*=114, *R*^2^_m_=0.105, *R*^2^_c_=0.366, *P*<0.001) and depth of stroke cessation (*N*=269, *R*^2^_m_=0.287, *R*^2^_c_=0.384, *P*<0.001) (Fig. 3, Table 2).

**Table 2.** Summary table of the relationships between DRAV and RMS sway, and DRAV and depth at stroke cessation

Parameter	Intercept	Gradient	*R* ^2^_m_	*R* ^2^_c_
RMS sway∼DRAV (*N*=114)	1.010±0.059	0.0066±0.0017*	0.105	0.366
corAR(1) coefficient: ϕ=0.243				
Depth of stroke cessation∼DRAV (*N*=269)	10.10±1.34	0.423±0.042*	0.287	0.384
corAR(1) coefficient: ϕ=0.335				

Intercept and gradient data are coefficient±s.e. *R*^2^_m_ represents the marginal *R*^2^ from the linear mixed model, accounting only for the variation from the fixed effect (DRAV). *R*^2^_c_ represents the conditional *R*^2^ from the model, accounting for the random effect (individual variation). The corAR(1) coefficient (ϕ) is reported for both models. Asterisks indicate statistical significance (*P<0.001).

**Fig. 4.**
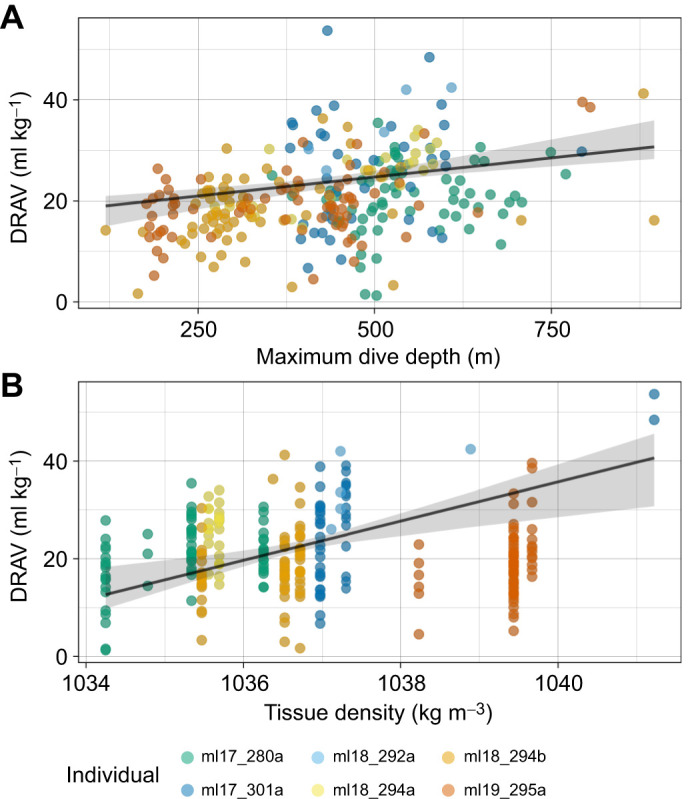
**The relationship of DRAV with maximum dive depth and tissue density.** (A) DRAV and maximum dive depth. (B) DRAV and tissue density. The line represents the estimates of the fixed effects from the linear mixed model, and the grey shading represents the 95% confidence intervals. The colours represent each individual, with one data point per dive. The fixed effects explained 30.9% of the variation in DRAV (*R*^2^_m_=0.309), while 68.4% of the variation was explained with both the fixed effects and individual variability (*R*^2^_c_=0.684). The autocorrelation coefficient for the corAR(1) structure was ϕ=0.340, suggesting that successive DRAV had a moderate positive autocorrelation.

To explore whether elephant seals might adjust their DRAV to reduce swimming costs, we examined the relationship between DRAV, maximum dive depth and tissue density (Fig. 4, Tables 3 and 4). The most parsimonious model included an interaction between dive depth and tissue density. However, ΔAIC between the interaction model and a simpler additive model was <2, indicating similar explanatory power. Additionally, dive depth and tissue density had the highest summed Akaike weights (AIC_w_=1 for both), while the Akaike weight for the interaction term was notably lower (AIC_w_=0.63). Based on these results, we selected the simpler additive model for further analysis (Table 3).

**Table 3.** Model fit for linear mixed models to data on DRAV where individual was stated as a random effect, indicated by Akaike's information criterion (AIC)

Model	AIC	ΔAIC
DRAV∼Null	1817.7	35.8
DRAV∼Maximum dive depth	1802.1	20.2
DRAV∼Tissue density	1798.7	16.8
DRAV∼Maximum dive depth+Tissue density	1783.1	1.2
DRAV∼Maximum dive depth×Tissue density	1781.9	–

An autocorrelation term corAR(1) was included in each model.

**Table 4.** Summary table of the selected linear mixed model with individual as a random effect and a corAR(1) correlation structure: DRAV∼Maximum dive depth+Tissue density

Predictors	Estimates	CI	*P*
**Fixed effects**			** **
Dive depth (m)	0.02	0.01–0.02	**<0.001**
Tissue density (kg m^−3^)	4.01	2.47–5.56	**<0.001**
corAR(1) coefficient (ϕ)	0.340	–	**–**
**Random effects**			** **
σ^2^	45.12		** **
*N*_individuals_	6		** **
Observations	269		** **
*R*^2^_m_	0.309		** **
*R*^2^_c_	0.684		** **

The estimates and confidence intervals (CI) for the variables of interest for each model have been included. Significant *P*-values are in bold.

## DISCUSSION

All six elephant seals in this study were negatively buoyant, making it possible to estimate DRAV while the seals were passively gliding during the descent phase at shallow depths. Mean DRAV (21.1 ml kg^−1^) exhibited substantial variability (coefficient of variation CV=0.38), reflecting both dive-to-dive and between-individual differences. The fixed effects (tissue density and maximum dive depth) explained 30.9% of the variation in DRAV (*R*^2^_m_=0.309). Specifically, the effect size of 0.02 for maximum dive depth indicates that DRAV increased by ∼2 ml kg^−1^ for every additional 100 m of dive depth. Additionally, DRAV increased by approximately 4 ml kg^−1^ for every unit increase in tissue density (Table 4). When both fixed and random effects were considered, the model explained 68.4% of the variation (*R*^2^_c_=0.684), indicating substantial individual-level differences in mean DRAV over repeated observations (Hertel et al., 2020).

The moderate autocorrelation observed in DRAV values [corAR(1) coefficient ϕ=0.34] suggests that seals exhibit a degree of dive-by-dive consistency in the amount of retained air volume. This probably reflects physiological strategy between successive dives: by maintaining relatively low DRAV and promoting early lung collapse, seals may cease pulmonary gas exchange early in the descent. This limits nitrogen absorption into blood and tissues, reducing nitrogen uptake and the risk of decompression sickness (Hooker et al., 2005). The consistency in DRAV across successive dives may thus reflect tight short-term control over respiratory air volume, especially during deep, repetitive diving when physiological risk is elevated (see Figs S1–S6). In contrast, broader variation in DRAV across all dives (CV=0.38) probably reflects differences in tissue density, dive depth, and potentially small adjustments in respiratory behaviour. Together, these findings suggest that DRAV is tightly regulated on short time scales to manage physiological risk, yet responsive to ecological and energetic factors over longer time scales.

### The effect of DRAV on swimming costs

Trassinelli (2016) proposed two mechanisms by which seals may adjust DRAV to reduce swimming costs: (1) seals may reduce DRAV as tissue density approaches neutral buoyancy; (2) seals may increase DRAV during deeper dives to achieve neutral buoyancy near mid-dive depth, which would minimise overall swimming effort. Our findings indicate that elephant seals adjust DRAV in response to both tissue density and maximum dive depth, consistent with Trassinelli's predictions (Fig. 4). Specifically, DRAV increased with both tissue density and maximum dive depth (Fig. 4), suggesting that elephant seals may fine-tune their buoyancy across different ecological contexts. Given that lung oxygen stores comprise only ∼4% of total oxygen stores in elephant seals (Ponganis et al., 2011), we conclude that adjustment of DRAV primarily benefits these divers by reducing swimming costs rather than maximizing oxygen storage.

**Fig. 5.**
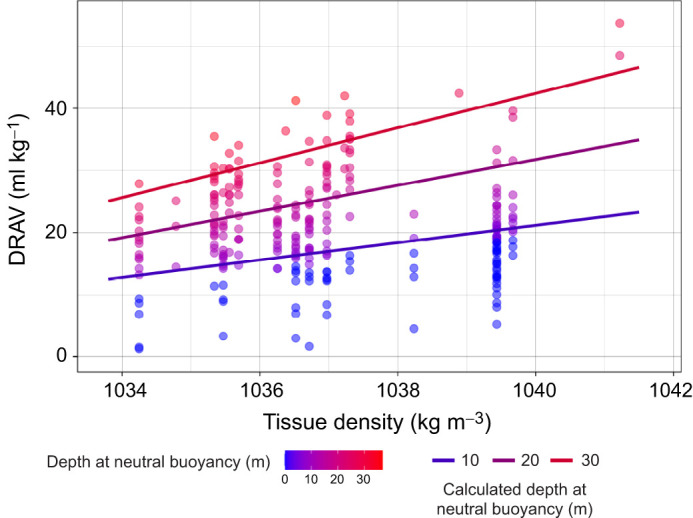
**The relationship between DRAV and tissue density.** Data are colour coded by the depth at which neutral buoyancy was achieved. The lines represent the calculated depth at neutral buoyancy achieved for a vertically swimming 300 kg individual, assuming that the sea water density is constant at 1025 kg m^−3^.

To further evaluate how DRAV influences swimming costs, we calculated the depth at which neutral buoyancy was achieved based on a representative range of DRAV and tissue density estimates. Fig. 5 illustrates the calculated neutral buoyancy depths for different net buoyancy values (sum of DRAV and tissue density). The lines indicate the achieved neutral buoyancy depths (10, 20 and 30 m), assuming vertical swimming [sin(pitch)=−1] in seawater of density 1025 kg m^−3^.

Trassinelli (2016) suggested that the optimal strategy for negatively buoyant animals would be to achieve neutral buoyancy at half the maximum dive depth. However, our results show that these seals typically would reach neutral buoyancy at less than 10% of their maximum dive depths, indicating that DRAV adjustments alone are insufficient to achieve this optimal strategy (see Fig. 5). For example, to reach neutral buoyancy at 200 m (half of a 400 m dive), a negatively buoyant seal weighing 417 kg with a tissue density of 1037.3 kg m^−3^ (such as seal ml17_301a on day 17) would need to maintain a DRAV of 247 ml kg^−1^. This far exceeds the highest estimated DRAV (53.7 ml kg^−1^) from this study and the measurements from forced submersion experiments (50.5 ml kg^−1^) by Kooyman et al. (1972).

These calculations highlight a physiological limit for negatively buoyant seals to adjust DRAV to reduce swimming costs. This has important implications for the behavioural plasticity of capital breeders like elephant seals, particularly in their ability to optimise energy use in a rapidly changing environment. Population models show that elephant seals are sensitive to changes in resource availability and energy demands, making them vulnerable to climate change and anthropogenic disturbances (Goedegebuure et al., 2018; New et al., 2014). As net buoyancy significantly affects round-trip swimming costs, understanding the behavioural plasticity in DRAV is especially important for non-neutrally buoyant individuals. However, our findings suggest that this plasticity is insufficient for negatively buoyant seals to achieve neutral buoyancy. As a result, individuals in poor body condition may be unable to fully offset the increased energetic costs through DRAV adjustment alone.

There is still limited information on DRAV adjustment among free-ranging divers. DRAV regulation could play a prominent role, especially in shallow-diving species, as the influence of gas on buoyancy is strongest at shallow depths. Future studies could explore how DRAV contributes to buoyancy control and energy expenditure in shallow-diving species, where the effect of gas on buoyancy is greatest.

### Sensitivity of DRAV estimates to the drag coefficient

Our overall mean (±s.d.) DRAV estimate was 21.1±8.1 ml kg^−1^. While these glide-based DRAV estimates align well with those from forced submersion experiments (mean 20.4 ml kg^−1^, range 10–55 ml kg^−1^; Kooyman et al., 1972), caution is needed when comparing them directly.

Estimates of DRAV from the hydrodynamic glide model depend on several parameters that were not directly measured. It is important to note that as these DRAV estimates are based on several estimated parameters in the model, the estimates have a level of uncertainty. However, these values represent the best available estimates of DRAV with current technology.

Sensitivity analyses demonstrated that varying *C*_d_ across the plausible range (0.02–0.05) affected the absolute values of DRAV (Table 5). Within a narrower range (0.02–0.04), kinematic correlations with DRAV remained significant. At higher *C*_d_ values, however, the increased contribution of drag to the deceleration rate reduced DRAV estimates and weakened the statistical relationship. Conversely, lower *C*_d_ values enhanced the relationship, as deceleration was more strongly attributed to buoyancy. Importantly, the overall results from the statistical model relating DRAV to maximum dive depth and tissue density remained unaffected by changes in *C*_d_, suggesting that the observed relationships are robust across a realistic range of *C*_d_.

**Table 5.** Sensitivity analysis of the effect of different drag coefficients on parameter estimates

*C*_d_	DRAV	DRAV∼Tissue density+Maximum dive depth			Depth of stroke cessation		RMS sway	
		*R* ^2^_m_	*P*_TD_	*P*_MD_	*R* ^2^_m_	*P*	*R* ^2^_m_	*P*
0.02	25.9±8.6	0.345	<0.001	<0.001	0.359	<0.001	0.134	<0.001
**0.03**	**21.1±8.1**	**0.309**	**<0.001**	**<0.001**	**0.287**	**<0.001**	**0.105**	**<0.001**
0.04	16.7±7.5	0.268	<0.001	<0.001	0.205	<0.001	0.079	0.002
0.05	12.3±7.2	0.221	<0.001	<0.001	0.118	<0.001	0.048	0.015

DRAV data are means±s.d. *C*_d_, drag coefficient; TD, tissue density; MD, maximum dive depth. *C*_d_=0.03 (marked in bold) was used in this study.

The model also assumes a perfectly compliant air space, where the trachea and lungs perfectly compress according to Boyle's law. Recent data from California sea lions suggest that compression may be less than predicted during deep dives (Cole et al., 2023), potentially leading to a systematic underestimation of DRAV at depth. However, deviations from Boyle's law should be smallest at shallower depths where the most compliant tissues collapse first. Given the inclusion of the compression correction factor in our model, giving greater statistical weight to shallower glides, we expect any deviation from perfect compliance to be small and accounted for in our analysis.

### Correlations between swimming effort and DRAV estimates

Statistically significant relationships between DRAV and both RMS dynamic acceleration and depth of stroke cessation, while accounting for individual variation, indicate that DRAV influences swimming kinematics and thrust production at shallow depths during descent. The consistency of these findings with our biomechanical predictions supports the conclusion that our method successfully captured dive-by-dive variation in DRAV.

However, caution is warranted when interpreting dynamic acceleration in terms of mechanical work. High-frequency dynamic acceleration reflects both specific acceleration (SA) and body rotations (BRs), and can be influenced by stroke amplitude, stroke frequency or both, complicating the relationship between acceleration and mechanical work performed (Martín López et al., 2015, 2016). Separating SA and BR using magnetometer data could improve biomechanical interpretations of thrust production during active stroking and support future studies of buoyancy-driven variation in swimming effort.

These biomechanical sources of variability probably contributed to the relatively low marginal *R*^2^ observed in our models. While DRAV significantly influenced kinematic parameters such as RMS sway acceleration and stroke cessation depth, these relationships explained a small proportion of the total variance. This is expected given that dynamic acceleration integrates multiple biomechanical components, such as stroke frequency, amplitude and BR, not all of which are driven solely by buoyancy or DRAV. Additionally, individual behavioural decisions may have introduced further unexplained variance in the kinematic signals. These factors collectively limit the explanatory power of DRAV alone to account for the full range of observed swimming effort across dives.

### Applicability of the method

Estimating DRAV from descent glides requires strict criteria, with only 18% of dives (269 of 1638) suitable for analysis (Table 1). Additionally, this method is unsuitable for positively buoyant individuals, as they tend to glide during ascent rather than descent (Adachi et al., 2014; Miller et al., 2016). This limitation reduces the available sample size, limiting insights into DRAV adjustments near neutral buoyancy.

Despite these limitations, DRAV estimates from descent glides are probably more reliable than those from ascent glides (Fahlman et al., 2020) because of potential sources of underestimation: (1) the influence of fuel sources for aerobic metabolism (i.e. glucose, fat, protein) on respiratory volume; and (2) the higher solubility of carbon dioxide relative to nitrogen and oxygen, resulting in an imbalance of gas diffusion rates across the lung–blood barrier during the dive, leading to differences in the number of molecules present inside the lungs (Fahlman et al., 2009; Weathersby and Homer, 1980). These factors could lead to an underestimation of DRAV by 12–50% for ascent glides (Fahlman et al., 2020).

Additionally, exhalations near the end of ascent, observed in divers such as penguins (Sato et al., 2011, 2002) and Antarctic fur seals (Hooker et al., 2005), could further affect DRAV estimates made during the ascent phase. However, this behaviour is not universal, and while it remains uncertain whether phocid seals exhale during ascent, current evidence suggests it is unlikely (Hooker et al., 2021).

Our methodology relies on estimating parameters such as tissue density and the drag coefficient using glides deeper than 100 m, a method currently feasible only for deep-diving species (Aoki et al., 2011). However, recent advances in hydrodynamic modelling have enabled speed change detection over 5 s sub-glides in both deep and shallow dives. This could potentially expand the applicability of DRAV estimation to a broader range of breath-hold divers, including shallow-diving species (Aoki et al., 2021; Narazaki et al., 2018).

## Supplementary Material

10.1242/jexbio.249659_sup1 Supplementary information
