# Supplementary material for: Dive-by-dive variation in the diving respiratory air volume of southern elephant seals (Mirounga leonina)
Source: J Exp Biol. 2025 May 23;228(10):jeb249659. doi: 10.1242/jeb.249659 (PMC12148017; doi:10.1242/jeb.249659)
Supplement: Supplementary information [file jexbio-228-249659-s1.pdf]

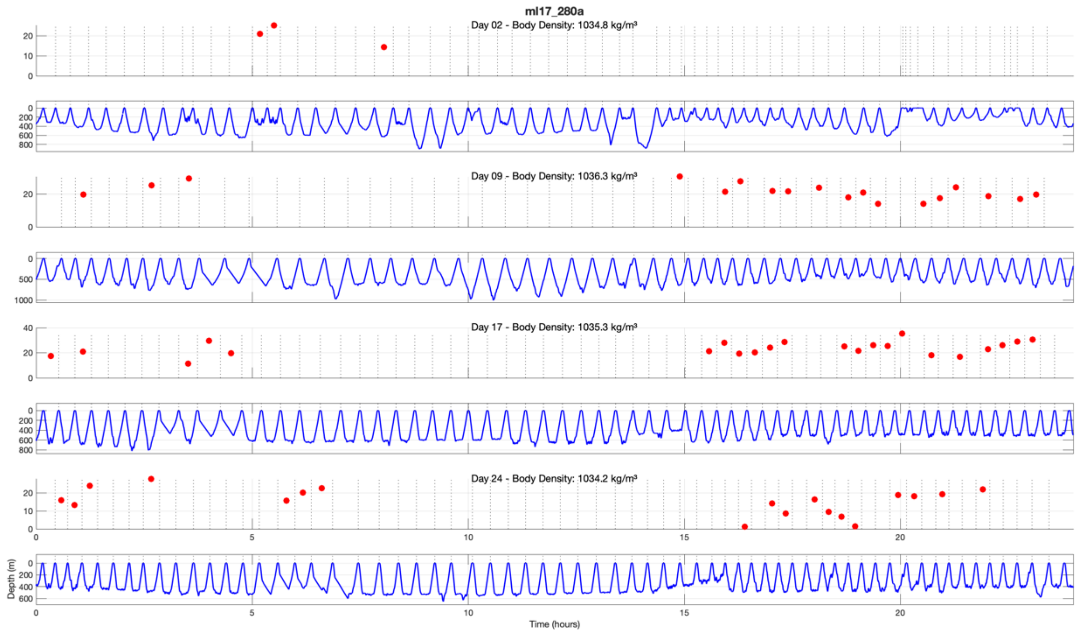

Fig. S1. Time series of DRAV (red points) and dive depth (blue line) for the individual ml17_280a across four days (Day 2, 9, 17 and 24). Each subplot consists of two parts. First row: Red scatter points represent the estimated DRAV. Second row: The blue line represents the dive depth.

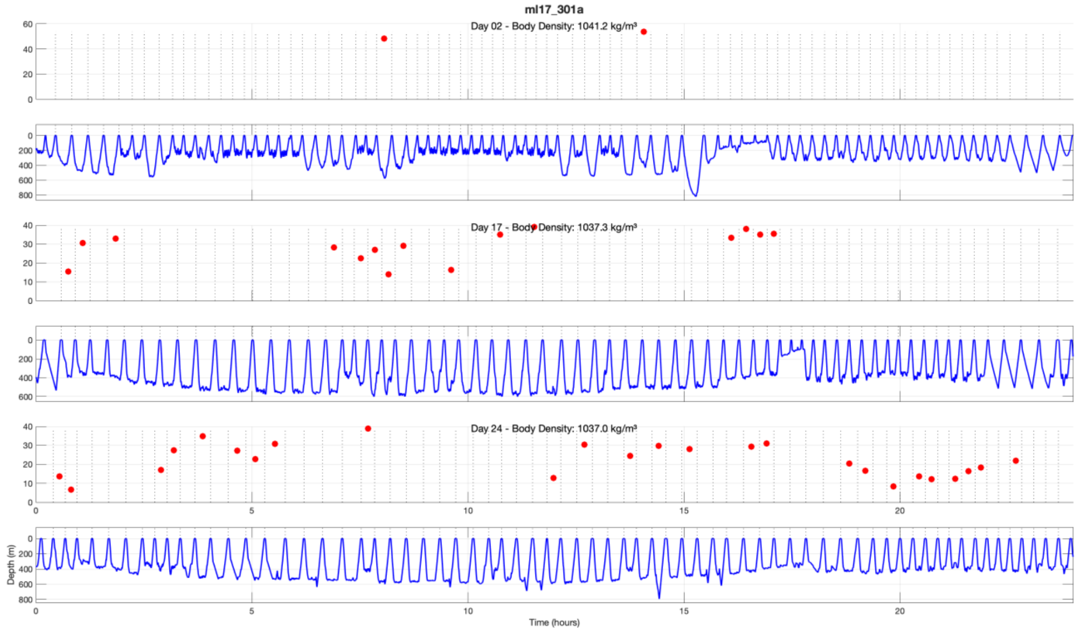

Fig. S2. Time series of DRAV (red points) and dive depth (blue line) for the individual ml17_301a across four days (Day 2, 9, 17 and 24).

Journal of Experimental Biology • Supplementary information

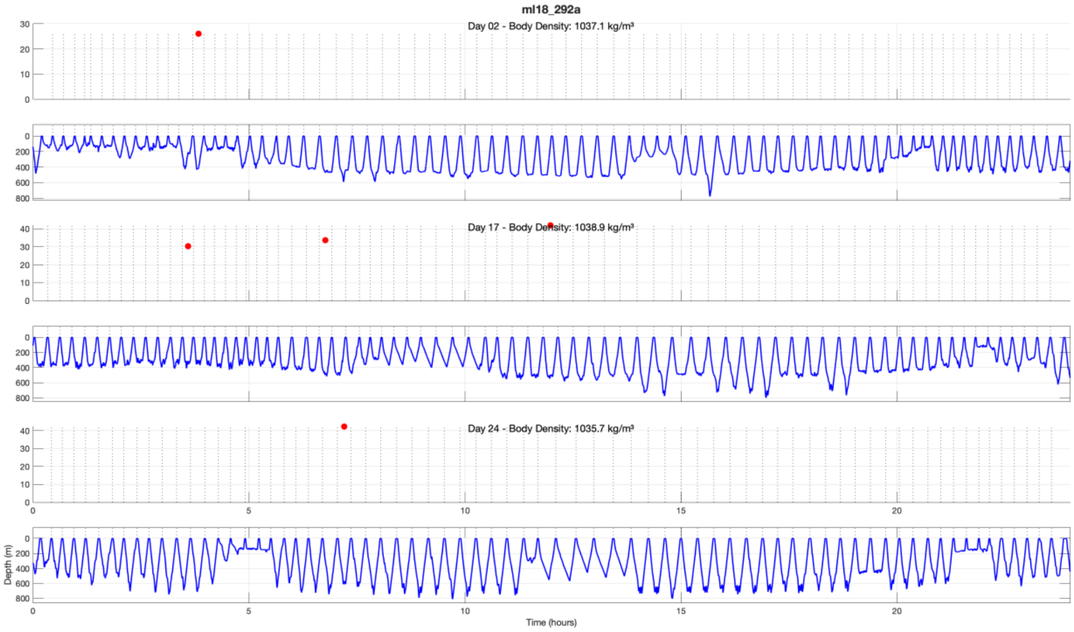

Fig. S3. Time series of DRAV (red points) and dive depth (blue line) for the individual ml18_292a across four days (Day 2, 9, 17 and 24).

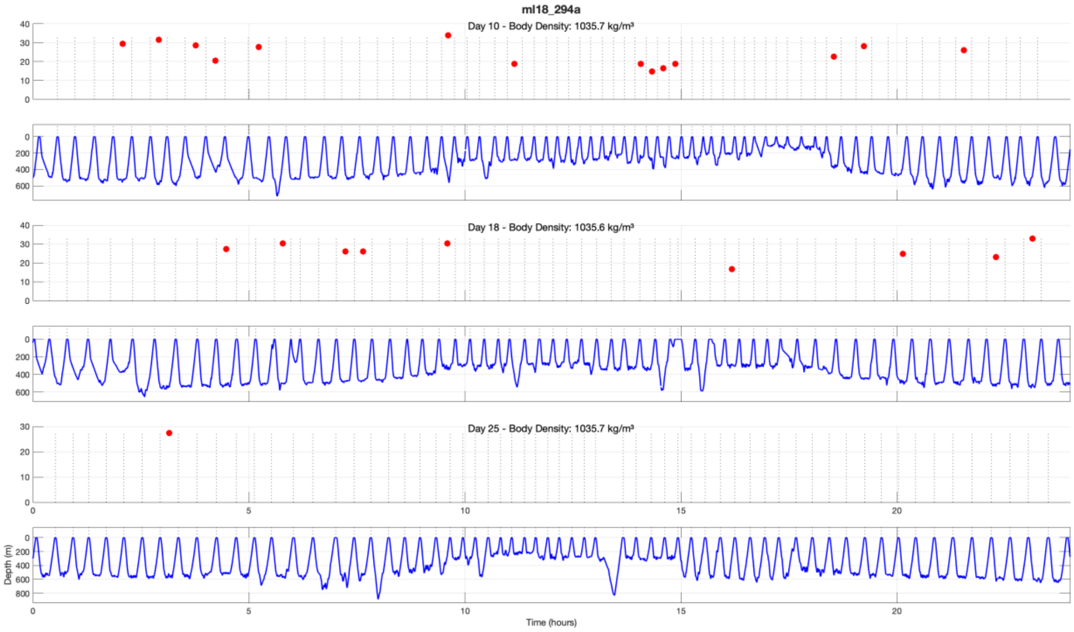

Fig. S4. Time series of DRAV (red points) and dive depth (blue line) for the individual ml18_294a across four days (Day 3, 10, 18 and 25).

Journal of Experimental Biology • Supplementary information

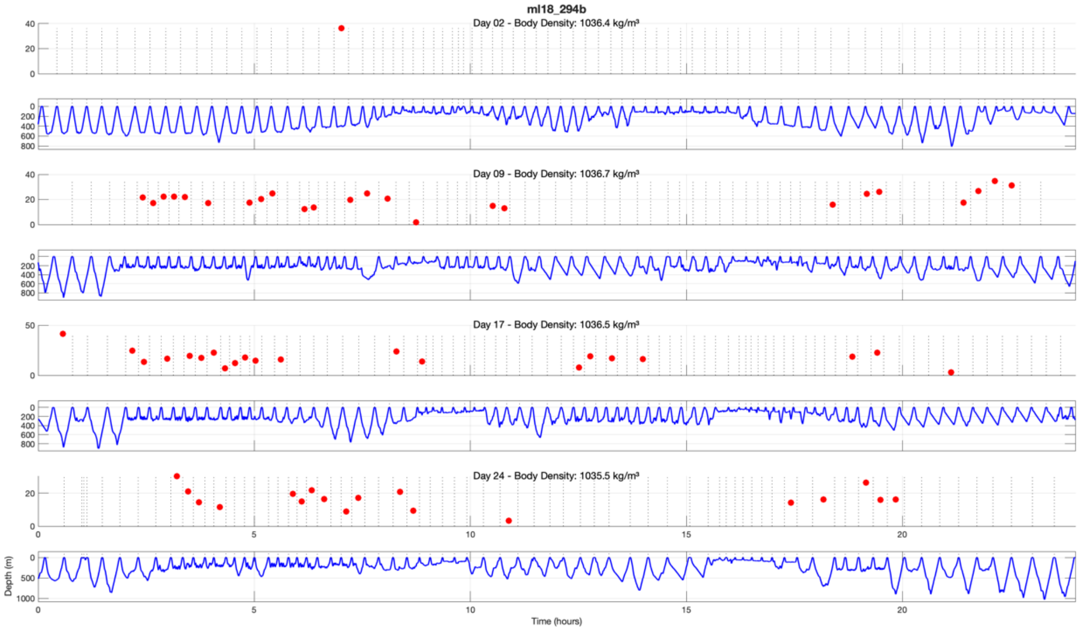

Fig. S5. Time series of DRAV (red points) and dive depth (blue line) for the individual ml18_294b across four days (Day 2, 9, 17 and 24).

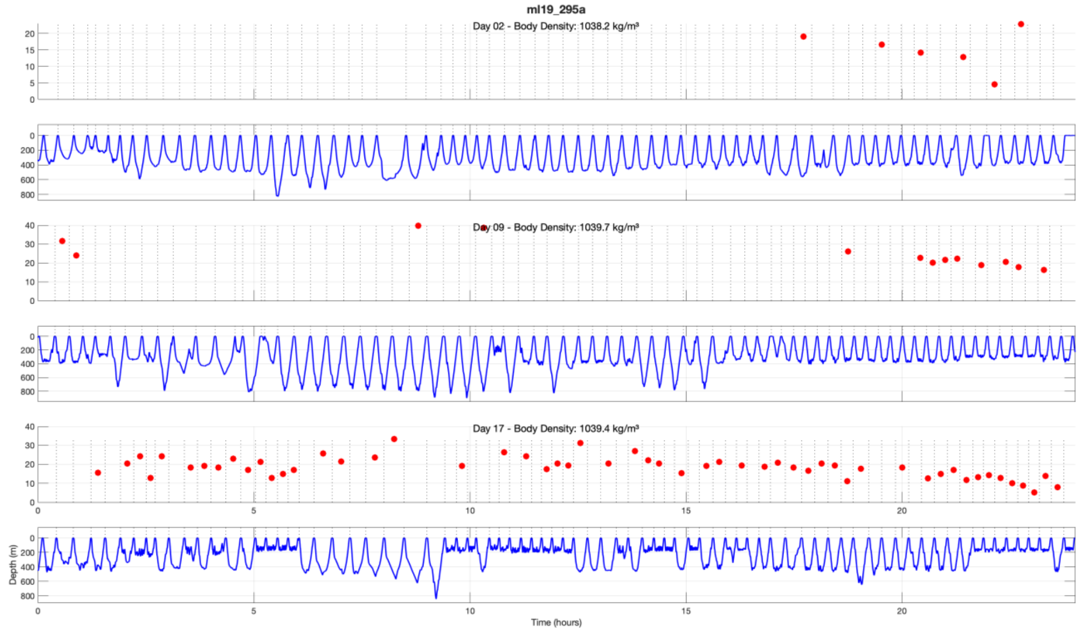

Fig. S6. Time series of DRAV (red points) and dive depth (blue line) for the individual ml19_295a across three days (Day 2, 9 and 17).
